# Supplementary material for: Analyses of Transcriptomics Cell Signalling for Pre-Screening Applications in the Integrated Approach for Testing and Assessment of Non-Genotoxic Carcinogens
Source: Int J Mol Sci. 2022 Oct 22;23(21):12718. doi: 10.3390/ijms232112718 (PMC9659232; doi:10.3390/ijms232112718)
Supplement: Supplementary file 1 [file ijms-23-12718-s001.zip › Supplementary_Figures_S1_S7 rev.pdf]

Figure S1

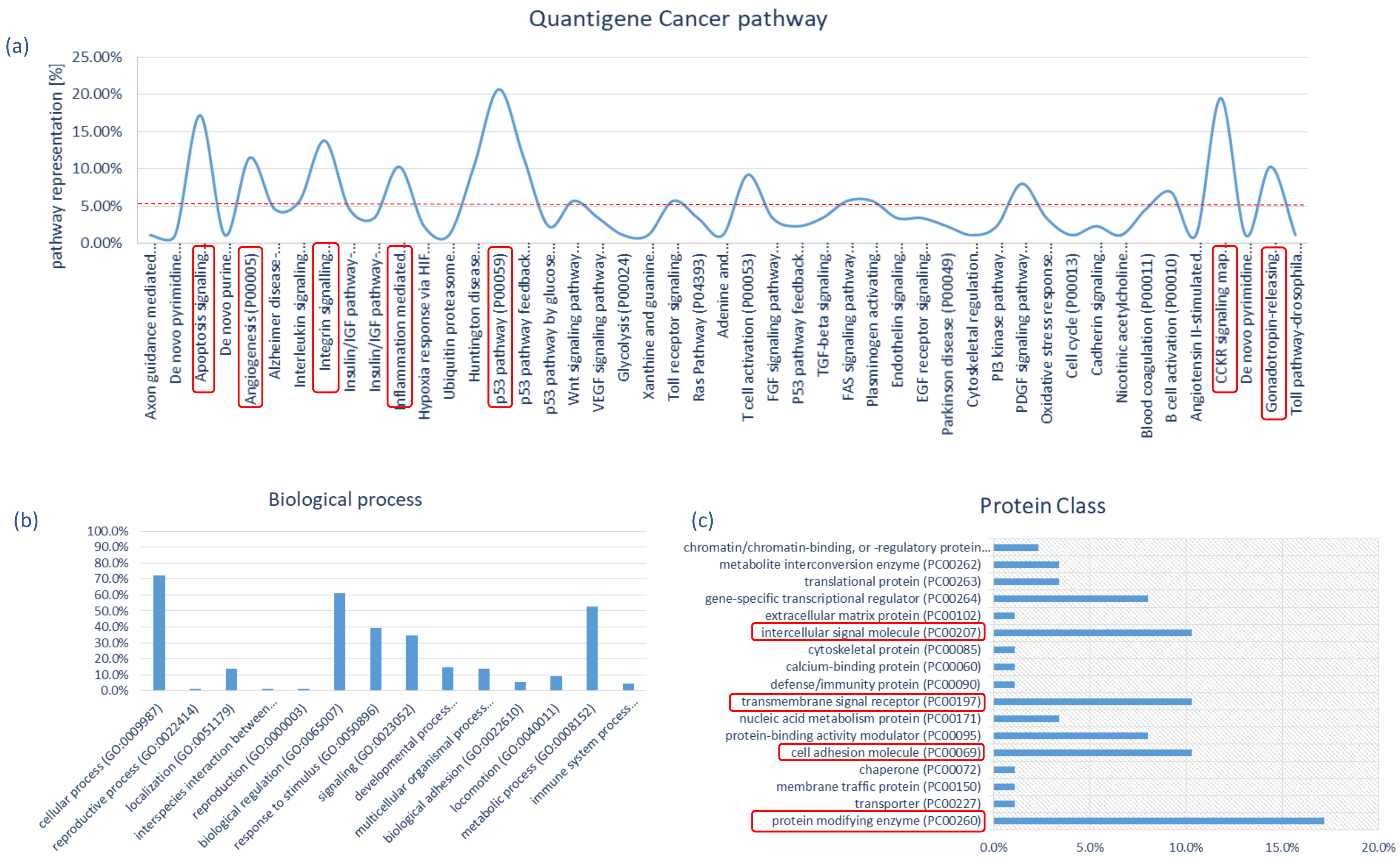

(a)
Quantigene Cancer pathway
pathway representation [%]
25.00%
20.00%
15.00%
10.00%
5.00%
0.00%
Axon guidance mediated...
De novo pyrimidine...
Apoptosis signaling...
De novo purine...
Angiogenesis (P00005)
Alzheimer disease-...
Interleukin signaling...
Integrin signalling...
Insulin/IGF pathway-...
Insulin/IGF pathway-...
Inflammation mediated...
Hypoxia response via HIF...
Ubiquitin proteasome...
Huntington disease...
p53 pathway (P00059)
p53 pathway feedback...
p53 pathway by glucose...
Wnt signaling pathway...
VEGF signaling pathway...
Glycolysis (P00024)
Xanthine and guanine...
Toll receptor signaling...
Ras Pathway (P04393)
Adenine and...
T cell activation (P00053)
FGF signaling pathway...
P53 pathway feedback...
TGF-beta signaling...
FAS signaling pathway...
Plasminogen activating...
Endothelin signaling...
EGF receptor signaling...
Parkinson disease (P00049)
Cytoskeletal regulation...
PI3 kinase pathway...
PDGF signaling pathway...
Oxidative stress response...
Cell cycle (P00013)
Cadherin signaling...
Nicotinic acetylcholine...
Blood coagulation (P00011)
B cell activation (P00010)
Angiotensin II-stimulated...
CCKR signaling map...
De novo pyrimidine...
Gonadotropin-releasing...
Toll pathway-drosophila...
(b)
Biological process
100.0%
90.0%
80.0%
70.0%
60.0%
50.0%
40.0%
30.0%
20.0%
10.0%
0.0%
cellular process (GO:0009987)
reproductive process (GO:0022414)
localization (GO:0051179)
interspecies interaction between...
reproduction (GO:0000003)
biological regulation (GO:0065007)
response to stimulus (GO:0050896)
signaling (GO:0023052)
developmental process...
multicellular organismal process...
biological adhesion (GO:0022610)
locomotion (GO:0040011)
metabolic process (GO:0008152)
immune system process...
(c)
Protein Class
chromatin/chromatin-binding, or -regulatory protein...
metabolite interconversion enzyme (PC00262)
translational protein (PC00263)
gene-specific transcriptional regulator (PC00264)
extracellular matrix protein (PC00102)
intercellular signal molecule (PC00207)
cytoskeletal protein (PC00085)
calcium-binding protein (PC00060)
defense/immunity protein (PC00090)
transmembrane signal receptor (PC00197)
nucleic acid metabolism protein (PC00171)
protein-binding activity modulator (PC00095)
cell adhesion molecule (PC00069)
chaperone (PC00072)
membrane traffic protein (PC00150)
transporter (PC00227)
protein modifying enzyme (PC00260)
0.0%
5.0%
10.0%
15.0%
20.0%

Figure S2

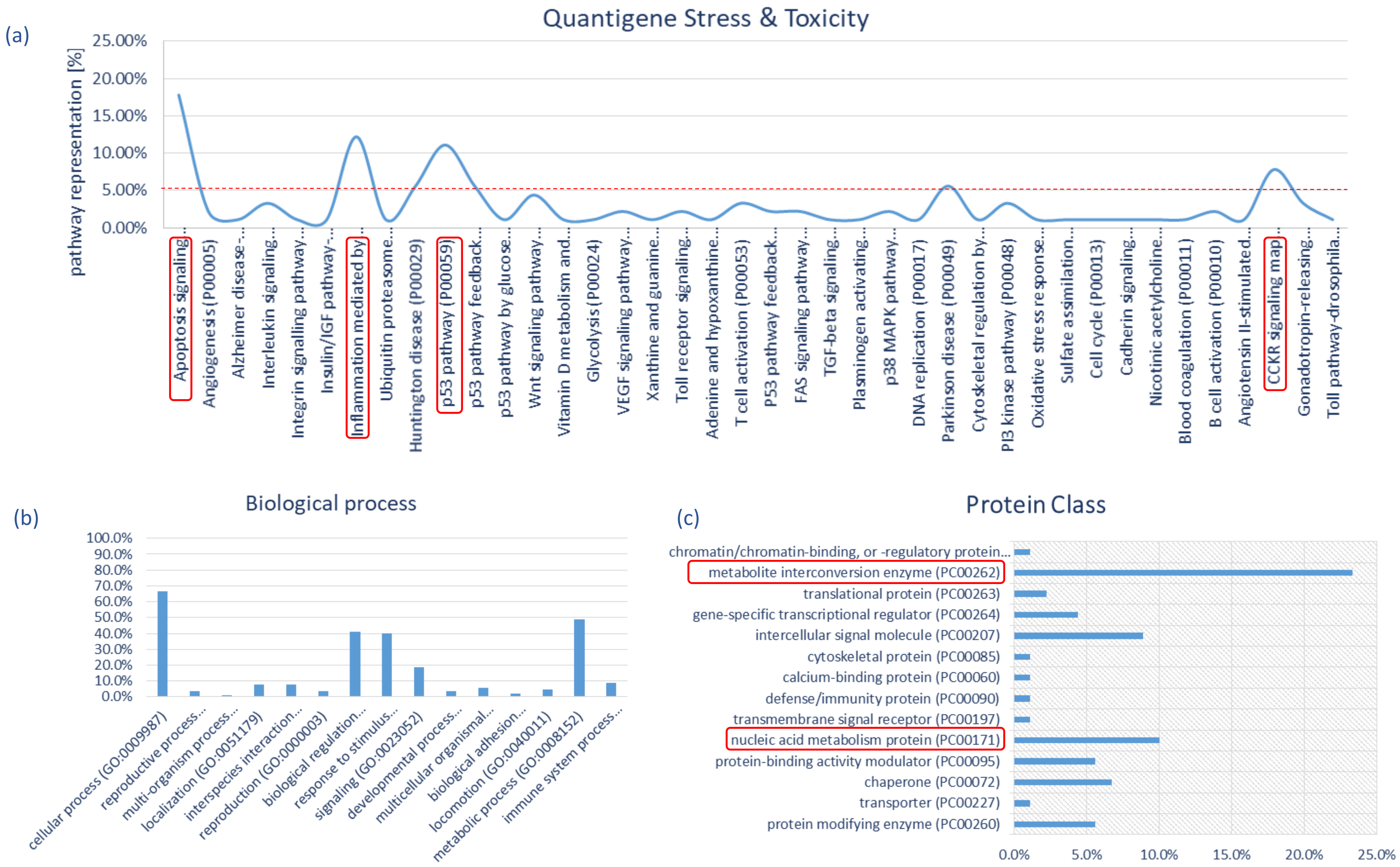
(a)
Quantigene Stress & Toxicity
pathway representation [%]
25.00%
20.00%
15.00%
10.00%
5.00%
0.00%
Apoptosis signaling...
Angiogenesis (P00005)
Alzheimer disease-...
Interleukin signaling...
Integrin signalling pathway...
Insulin/IGF pathway-...
Inflammation mediated by...
Ubiquitin proteasome...
Huntington disease (P00029)
p53 pathway (P00059)
p53 pathway feedback...
p53 pathway by glucose...
Wnt signaling pathway...
Vitamin D metabolism and...
Glycolysis (P00024)
VEGF signaling pathway...
Xanthine and guanine...
Toll receptor signaling...
Adenine and hypoxanthine...
T cell activation (P00053)
P53 pathway feedback...
FAS signaling pathway...
TGF-beta signaling...
Plasminogen activating...
p38 MAPK pathway...
DNA replication (P00017)
Parkinson disease (P00049)
Cytoskeletal regulation by...
PI3 kinase pathway (P00048)
Oxidative stress response...
Sulfate assimilation...
Cell cycle (P00013)
Cadherin signaling...
Nicotinic acetylcholine...
Blood coagulation (P00011)
B cell activation (P00010)
Angiotensin II-stimulated...
CCKR signaling map...
Gonadotropin-releasing...
Toll pathway-drosophila...
(b)
Biological process
100.0%
90.0%
80.0%
70.0%
60.0%
50.0%
40.0%
30.0%
20.0%
10.0%
0.0%
cellular process (GO:0009987)
reproductive process...
multi-organism process...
localization (GO:0051179)
interspecies interaction...
reproduction (GO:0000003)
biological regulation...
response to stimulus...
signaling (GO:0023052)
developmental process...
multicellular organismal...
biological adhesion...
locomotion (GO:0040011)
metabolic process (GO:0008152)
immune system process...
(c)
Protein Class
chromatin/chromatin-binding, or -regulatory protein...
metabolite interconversion enzyme (PC00262)
translational protein (PC00263)
gene-specific transcriptional regulator (PC00264)
intercellular signal molecule (PC00207)
cytoskeletal protein (PC00085)
calcium-binding protein (PC00060)
defense/immunity protein (PC00090)
transmembrane signal receptor (PC00197)
nucleic acid metabolism protein (PC00171)
protein-binding activity modulator (PC00095)
chaperone (PC00072)
transporter (PC00227)
protein modifying enzyme (PC00260)
0.0%
5.0%
10.0%
15.0%
20.0%
25.0%

Figure S3

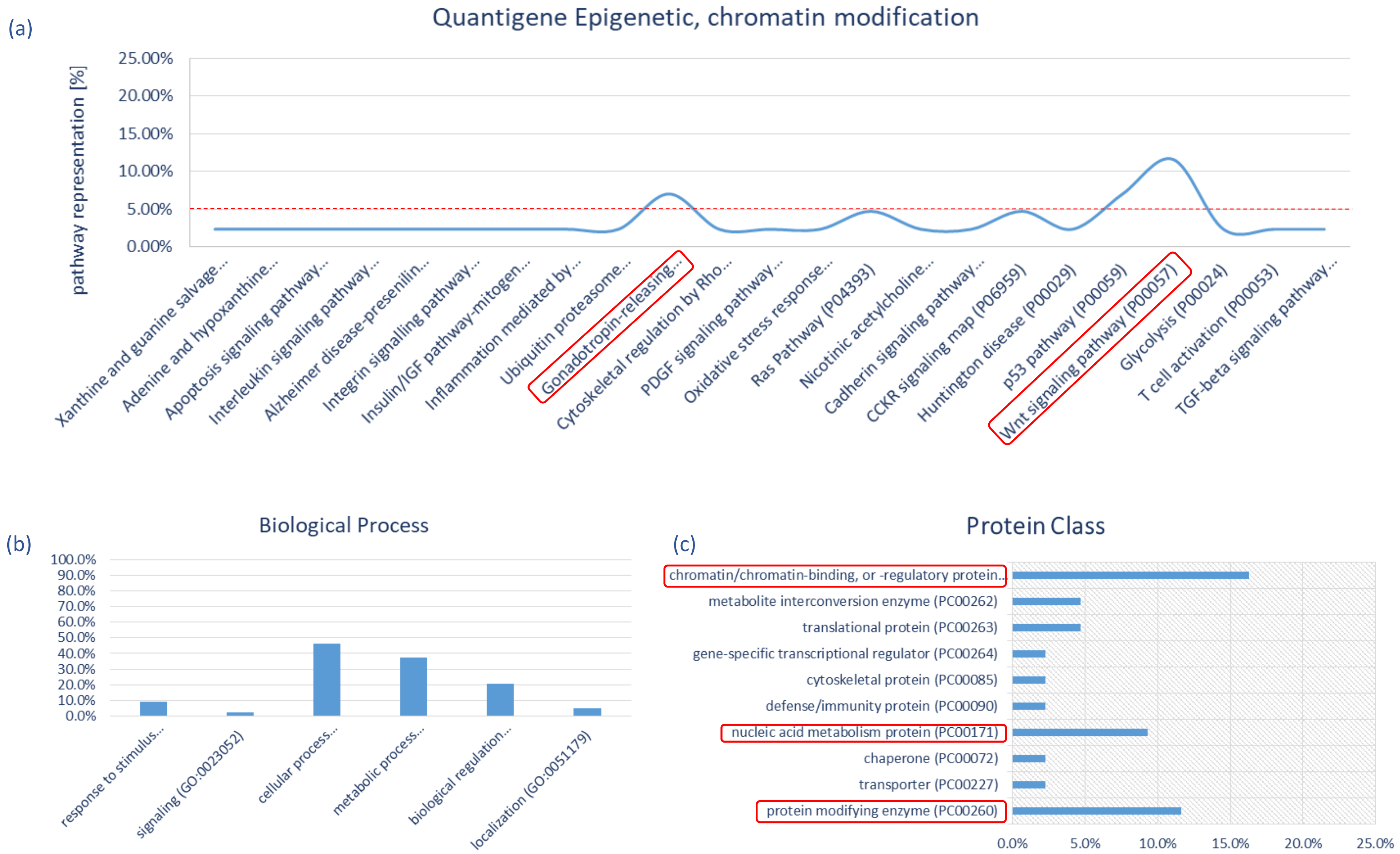

(a)
Quantigene Epigenetic, chromatin modification
pathway representation [%]
25.00%
20.00%
15.00%
10.00%
5.00%
0.00%
Xanthine and guanine salvage...
Adenine and hypoxanthine...
Apoptosis signaling pathway...
Interleukin signaling pathway...
Alzheimer disease-presenilin...
Integrin signalling pathway...
Insulin/IGF pathway-mitogen...
Inflammation mediated by...
Ubiquitin proteasome...
Gonadotropin-releasing...
Cytoskeletal regulation by Rho...
PDGF signaling pathway...
Oxidative stress response...
Ras Pathway (P04393)
Nicotinic acetylcholine...
Cadherin signaling pathway...
CCKR signaling map (P06959)
Huntington disease (P00029)
p53 pathway (P00059)
Wnt signaling pathway (P00057)
Glycolysis (P00024)
T cell activation (P00053)
TGF-beta signaling pathway...
(b)
Biological Process
100.0%
90.0%
80.0%
70.0%
60.0%
50.0%
40.0%
30.0%
20.0%
10.0%
0.0%
response to stimulus...
signaling (GO:0023052)
cellular process...
metabolic process...
biological regulation...
localization (GO:0051179)
(c)
Protein Class
chromatin/chromatin-binding, or -regulatory protein...
metabolite interconversion enzyme (PC00262)
translational protein (PC00263)
gene-specific transcriptional regulator (PC00264)
cytoskeletal protein (PC00085)
defense/immunity protein (PC00090)
nucleic acid metabolism protein (PC00171)
chaperone (PC00072)
transporter (PC00227)
protein modifying enzyme (PC00260)
0.0%
5.0%
10.0%
15.0%
20.0%
25.0%

Figure S4

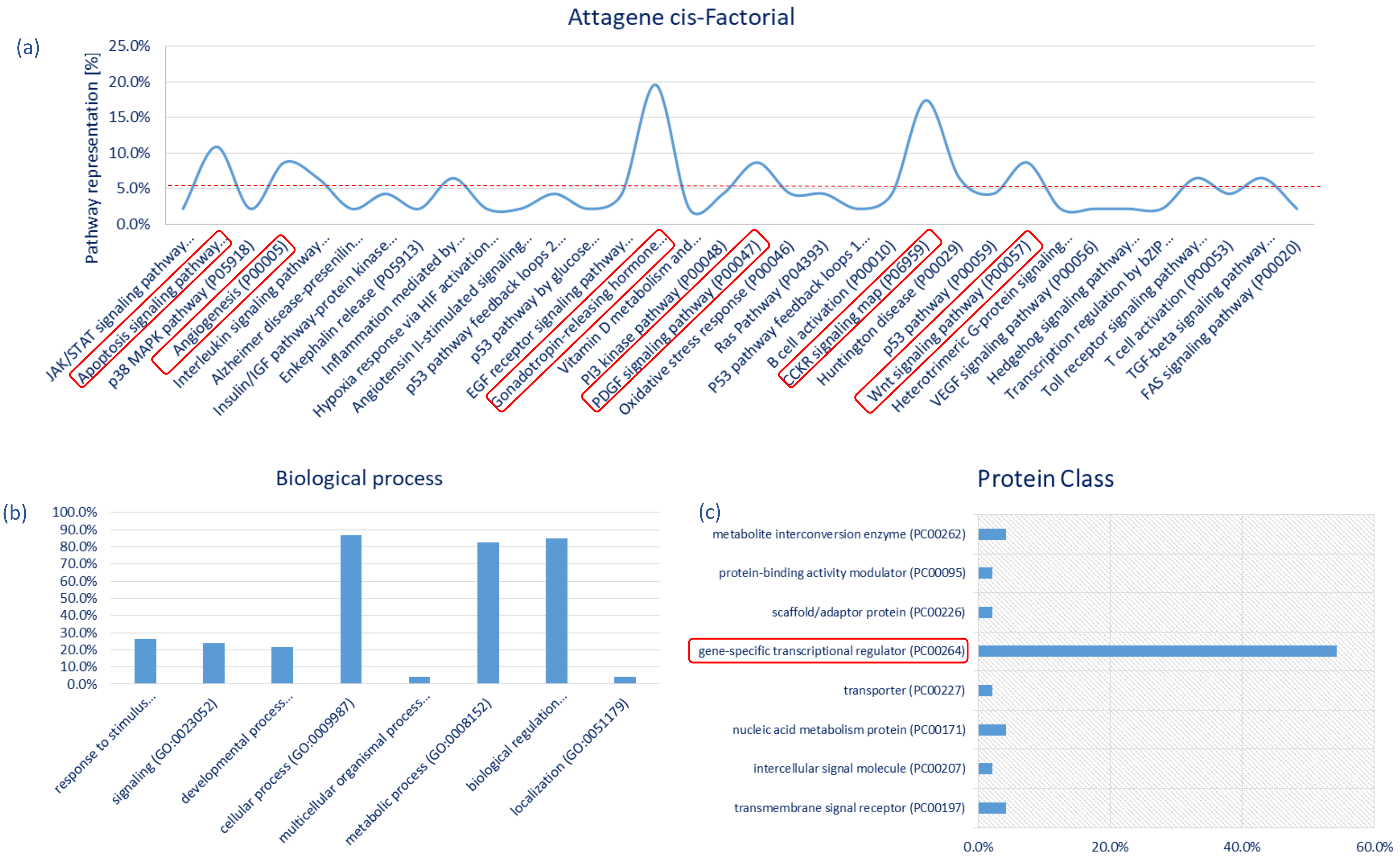
Attagene cis-Factorial
(a)
Pathway representation [%]
25.0%
20.0%
15.0%
10.0%
5.0%
0.0%
JAK/STAT signaling pathway...
Apoptosis signaling pathway...
p38 MAPK pathway (P05918)
Angiogenesis (P00005)
Interleukin signaling pathway...
Alzheimer disease-presenilin...
Insulin/IGF pathway-protein kinase...
Enkephalin release (P05913)
Inflammation mediated by...
Hypoxia response via HIF activation...
Angiotensin II-stimulated signaling...
p53 pathway feedback loops 2...
p53 pathway by glucose...
EGF receptor signaling pathway...
Gonadotropin-releasing hormone...
Vitamin D metabolism and...
PI3 kinase pathway (P00048)
PDGF signaling pathway (P00047)
Oxidative stress response (P00046)
Ras Pathway (P04393)
P53 pathway feedback loops 1...
B cell activation (P00010)
CCKR signaling map (P06959)
Huntington disease (P00029)
p53 pathway (P00059)
Wnt signaling pathway (P00057)
Heterotrimeric G-protein signaling...
VEGF signaling pathway (P00056)
Hedgehog signaling pathway...
Transcription regulation by bZIP...
Toll receptor signaling pathway...
T cell activation (P00053)
TGF-beta signaling pathway...
FAS signaling pathway (P00020)
Biological process
(b)
100.0%
90.0%
80.0%
70.0%
60.0%
50.0%
40.0%
30.0%
20.0%
10.0%
0.0%
response to stimulus...
signaling (GO:0023052)
developmental process...
cellular process (GO:0009987)
multicellular organismal process...
metabolic process (GO:0008152)
biological regulation...
localization (GO:0051179)
Protein Class
(c)
metabolite interconversion enzyme (PC00262)
protein-binding activity modulator (PC00095)
scaffold/adaptor protein (PC00226)
gene-specific transcriptional regulator (PC00264)
transporter (PC00227)
nucleic acid metabolism protein (PC00171)
intercellular signal molecule (PC00207)
transmembrane signal receptor (PC00197)
0.0%
20.0%
40.0%
60.0%

Figure S5

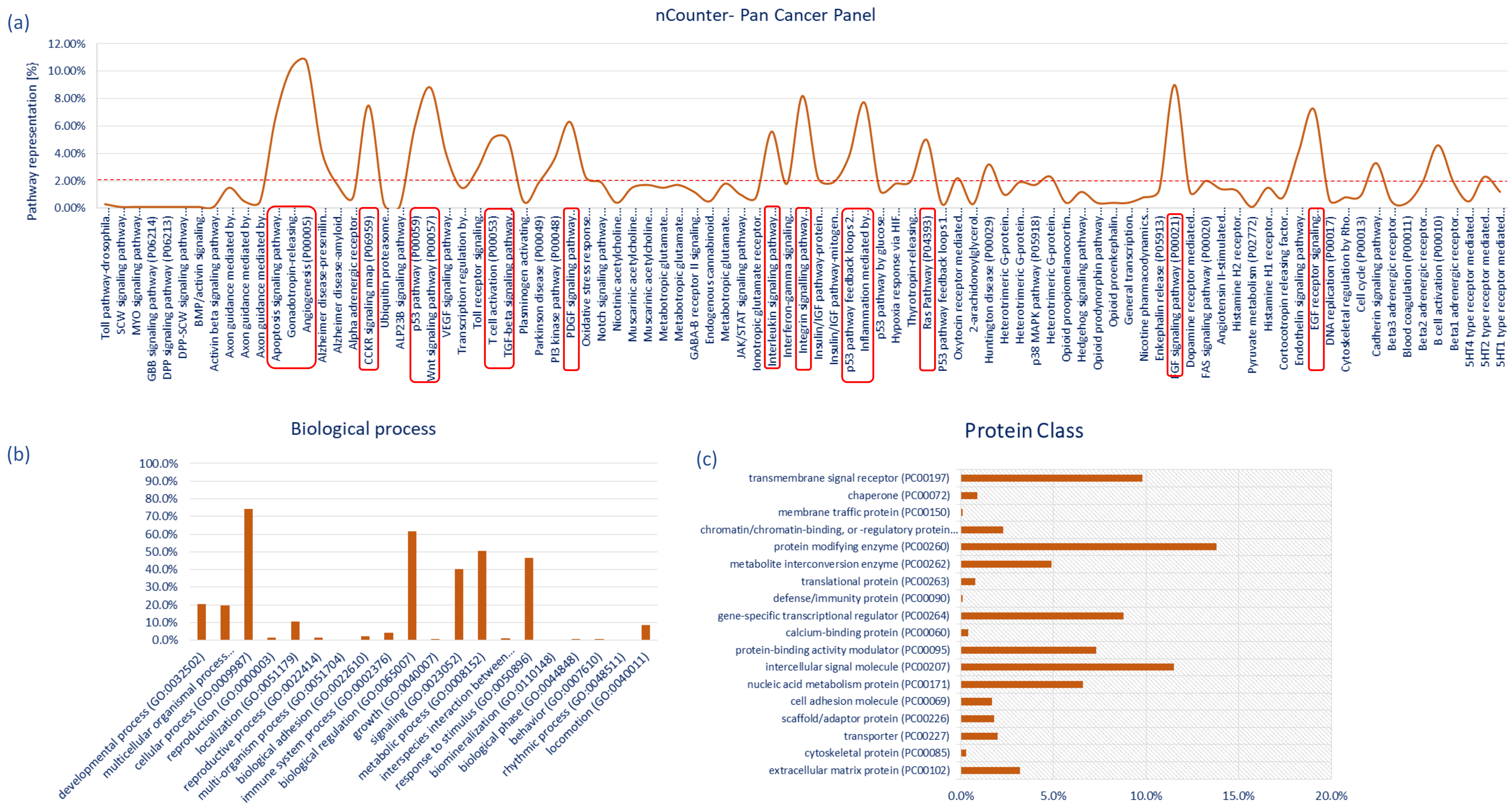
(a)
nCounter- Pan Cancer Panel
Pathway representation [%]
12.00%
10.00%
8.00%
6.00%
4.00%
2.00%
0.00%
Toll pathway-drosophila...
SCW signaling pathway...
MYO signaling pathway...
GBB signaling pathway (P06214)
DPP signaling pathway (P06213)
DPP-SCW signaling pathway...
BMP/activin signaling...
Activin beta signaling pathway...
Axon guidance mediated by...
Axon guidance mediated by...
Axon guidance mediated by...
Apoptosis signaling pathway...
Gonadotropin-releasing...
Angiogenesis (P00005)
Alzheimer disease-presenilin...
Alzheimer disease-amyloid...
Alpha adrenergic receptor...
CCKR signaling map (P06959)
Ubiquitin proteasome...
ALP23B signaling pathway...
p53 pathway (P00059)
Wnt signaling pathway (P00057)
VEGF signaling pathway...
Transcription regulation by...
Toll receptor signaling...
T cell activation (P00053)
TGF-beta signaling pathway...
Plasminogen activating...
Parkinson disease (P00049)
PI3 kinase pathway (P00048)
PDGF signaling pathway...
Oxidative stress response...
Notch signaling pathway...
Nicotinic acetylcholine...
Muscarinic acetylcholine...
Muscarinic acetylcholine...
Metabotropic glutamate...
Metabotropic glutamate...
GABA-B receptor II signaling...
Endogenous cannabinoid...
Metabotropic glutamate...
JAK/STAT signaling pathway...
Ionotropic glutamate receptor...
Interleukin signaling pathway...
Interferon-gamma signaling...
Integrin signalling pathway...
Insulin/IGF pathway-protein...
Insulin/IGF pathway-mitogen...
p53 pathway feedback loops 2...
Inflammation mediated by...
p53 pathway by glucose...
Hypoxia response via HIF...
Thyrotropin-releasing...
Ras Pathway (P04393)
P53 pathway feedback loops 1...
Oxytocin receptor mediated...
2-arachidonoylglycerol...
Huntington disease (P00029)
Heterotrimeric G-protein...
Heterotrimeric G-protein...
p38 MAPK pathway (P05918)
Heterotrimeric G-protein...
Opioid proopiomelanocortin...
Hedgehog signaling pathway...
Opioid prodynorphin pathway...
Opioid proenkephalin...
General transcription...
Nicotine pharmacodynamics...
Enkephalin release (P05913)
FGF signaling pathway (P00021)
Dopamine receptor mediated...
FAS signaling pathway (P00020)
Angiotensin II-stimulated...
Histamine H2 receptor...
Pyruvate metabolism (P02772)
Histamine H1 receptor...
Cortocotropin releasing factor...
Endothelin signaling pathway...
EGF receptor signaling...
DNA replication (P00017)
Cytoskeletal regulation by Rho...
Cell cycle (P00013)
Cadherin signaling pathway...
Beta3 adrenergic receptor...
Blood coagulation (P00011)
Beta2 adrenergic receptor...
B cell activation (P00010)
Beta1 adrenergic receptor...
5HT4 type receptor mediated...
5HT2 type receptor mediated...
5HT1 type receptor mediated...
(b)
Biological process
100.0%
90.0%
80.0%
70.0%
60.0%
50.0%
40.0%
30.0%
20.0%
10.0%
0.0%
developmental process (GO:0032502)
multicellular organismal process...
cellular process (GO:0009987)
reproduction (GO:0000003)
localization (GO:0051179)
reproductive process (GO:0022414)
multi-organism process (GO:0051704)
biological adhesion (GO:0022610)
immune system process (GO:0002376)
biological regulation (GO:0065007)
growth (GO:0040007)
signaling (GO:0023052)
metabolic process (GO:0008152)
interspecies interaction between...
response to stimulus (GO:0050896)
biomineralization (GO:0110148)
biological phase (GO:0044848)
behavior (GO:0007610)
rhythmic process (GO:0048511)
locomotion (GO:0040011)
(c)
Protein Class
transmembrane signal receptor (PC00197)
chaperone (PC00072)
membrane traffic protein (PC00150)
chromatin/chromatin-binding, or -regulatory protein...
protein modifying enzyme (PC00260)
metabolite interconversion enzyme (PC00262)
translational protein (PC00263)
defense/immunity protein (PC00090)
gene-specific transcriptional regulator (PC00264)
calcium-binding protein (PC00060)
protein-binding activity modulator (PC00095)
intercellular signal molecule (PC00207)
nucleic acid metabolism protein (PC00171)
cell adhesion molecule (PC00069)
scaffold/adaptor protein (PC00226)
transporter (PC00227)
cytoskeletal protein (PC00085)
extracellular matrix protein (PC00102)
0.0%
5.0%
10.0%
15.0%
20.0%

Figure S6

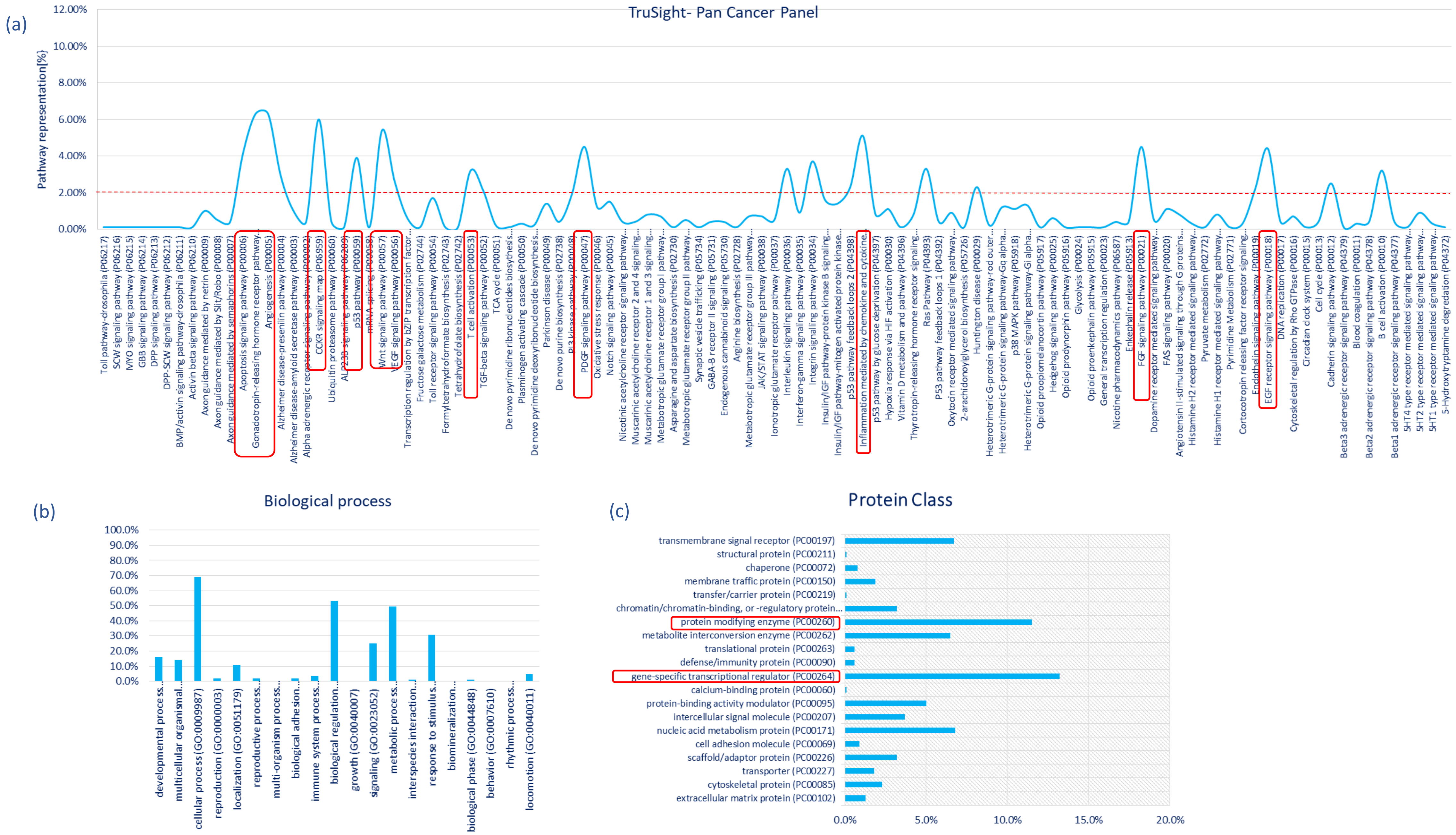
(a)
TruSight- Pan Cancer Panel
Pathway representation[%]
12.00%
10.00%
8.00%
6.00%
4.00%
2.00%
0.00%
Toll pathway-drosophila (P06217)
SCW signaling pathway (P06216)
MYO signaling pathway (P06215)
GBB signaling pathway (P06214)
DPP signaling pathway (P06213)
DPP-SCW signaling pathway (P06212)
BMP/activin signaling pathway-drosophila (P06211)
Activin beta signaling pathway (P06210)
Axon guidance mediated by netrin (P00009)
Axon guidance mediated by Slit/Robo (P00008)
Axon guidance mediated by semaphorins (P00007)
Apoptosis signaling pathway (P00006)
Gonadotropin-releasing hormone receptor pathway...
Angiogenesis (P00005)
Alzheimer disease-presenilin pathway (P00004)
Alzheimer disease-amyloid secretase pathway (P00003)
Alpha adrenergic receptor signaling pathway (P00002)
CCKR signaling map (P06959)
Ubiquitin proteasome pathway (P00060)
p53 pathway (P00059)
mRNA splicing (P00058)
Wnt signaling pathway (P00057)
VEGF signaling pathway (P00056)
Transcription regulation by bZIP transcription factor...
Fructose galactose metabolism (P02744)
Toll receptor signaling pathway (P00054)
Formyltetrahydroformate biosynthesis (P02743)
Tetrahydrofolate biosynthesis (P02742)
T cell activation (P00053)
TGF-beta signaling pathway (P00052)
TCA cycle (P00051)
De novo pyrimidine ribonucleotides biosynthesis...
Plasminogen activating cascade (P00050)
De novo pyrimidine deoxyribonucleotide biosynthesis...
Parkinson disease (P00049)
De novo purine biosynthesis (P02738)
PI3 kinase pathway (P00048)
PDGF signaling pathway (P00047)
Oxidative stress response (P00046)
Notch signaling pathway (P00045)
Nicotinic acetylcholine receptor signaling pathway...
Muscarinic acetylcholine receptor 2 and 4 signaling...
Muscarinic acetylcholine receptor 1 and 3 signaling...
Metabotropic glutamate receptor group I pathway...
Asparagine and aspartate biosynthesis (P02730)
Metabotropic glutamate receptor group II pathway...
Synaptic vesicle trafficking (P05734)
GABA-B receptor II signaling (P05731)
Endogenous cannabinoid signaling (P05730)
Arginine biosynthesis (P02728)
Metabotropic glutamate receptor group III pathway...
JAK/STAT signaling pathway (P00038)
Ionotropic glutamate receptor pathway (P00037)
Interleukin signaling pathway (P00036)
Interferon-gamma signaling pathway (P00035)
Integrin signalling pathway (P00034)
Insulin/IGF pathway-protein kinase B signaling...
Insulin/IGF pathway-mitogen activated protein kinase...
p53 pathway feedback loops 2 (P04398)
Inflammation mediated by chemokine and cytokine...
p53 pathway by glucose deprivation (P04397)
Hypoxia response via HIF activation (P00030)
Vitamin D metabolism and pathway (P04396)
Thyrotropin-releasing hormone receptor signaling...
Ras Pathway (P04393)
P53 pathway feedback loops 1 (P04392)
Oxytocin receptor mediated signaling pathway...
2-arachidonoylglycerol biosynthesis (P05726)
Huntington disease (P00029)
Heterotrimeric G-protein signaling pathway-rod outer...
Heterotrimeric G-protein signaling pathway-Gq alpha...
p38 MAPK pathway (P05918)
Heterotrimeric G-protein signaling pathway-Gi alpha...
Opioid proopiomelanocortin pathway (P05917)
Hedgehog signaling pathway (P00025)
Opioid prodynorphin pathway (P05916)
Glycolysis (P00024)
Opioid proenkephalin pathway (P05915)
General transcription regulation (P00023)
Nicotine pharmacodynamics pathway (P06587)
Enkephalin release (P05913)
FGF signaling pathway (P00021)
Dopamine receptor mediated signaling pathway...
FAS signaling pathway (P00020)
Angiotensin II-stimulated signaling through G proteins...
Histamine H2 receptor mediated signaling pathway...
Pyruvate metabolism (P02772)
Histamine H1 receptor mediated signaling pathway...
Pyrimidine Metabolism (P02771)
Cortocotropin releasing factor receptor signaling...
Endothelin signaling pathway (P00019)
EGF receptor signaling pathway (P00018)
DNA replication (P00017)
Cytoskeletal regulation by Rho GTPase (P00016)
Circadian clock system (P00015)
Cell cycle (P00013)
Cadherin signaling pathway (P00012)
Beta3 adrenergic receptor signaling pathway (P04379)
Blood coagulation (P00011)
Beta2 adrenergic receptor signaling pathway (P04378)
B cell activation (P00010)
Beta1 adrenergic receptor signaling pathway (P04377)
5HT4 type receptor mediated signaling pathway...
5HT2 type receptor mediated signaling pathway...
5HT1 type receptor mediated signaling pathway...
5-Hydroxytryptamine degredation (P04372)
(b)
Biological process
100.0%
90.0%
80.0%
70.0%
60.0%
50.0%
40.0%
30.0%
20.0%
10.0%
0.0%
developmental process...
multicellular organismal...
cellular process (GO:0009987)
reproduction (GO:0000003)
localization (GO:0051179)
reproductive process...
multi-organism process...
biological adhesion...
immune system process...
biological regulation...
growth (GO:0040007)
signaling (GO:0023052)
metabolic process...
interspecies interaction...
response to stimulus...
biomineralization...
biological phase (GO:0044848)
behavior (GO:0007610)
rhythmic process...
locomotion (GO:0040011)
(c)
Protein Class
transmembrane signal receptor (PC00197)
structural protein (PC00211)
chaperone (PC00072)
membrane traffic protein (PC00150)
transfer/carrier protein (PC00219)
chromatin/chromatin-binding, or -regulatory protein...
protein modifying enzyme (PC00260)
metabolite interconversion enzyme (PC00262)
translational protein (PC00263)
defense/immunity protein (PC00090)
gene-specific transcriptional regulator (PC00264)
calcium-binding protein (PC00060)
protein-binding activity modulator (PC00095)
intercellular signal molecule (PC00207)
nucleic acid metabolism protein (PC00171)
cell adhesion molecule (PC00069)
scaffold/adaptor protein (PC00226)
transporter (PC00227)
cytoskeletal protein (PC00085)
extracellular matrix protein (PC00102)
0.0%
5.0%
10.0%
15.0%
20.0%

Figure S7

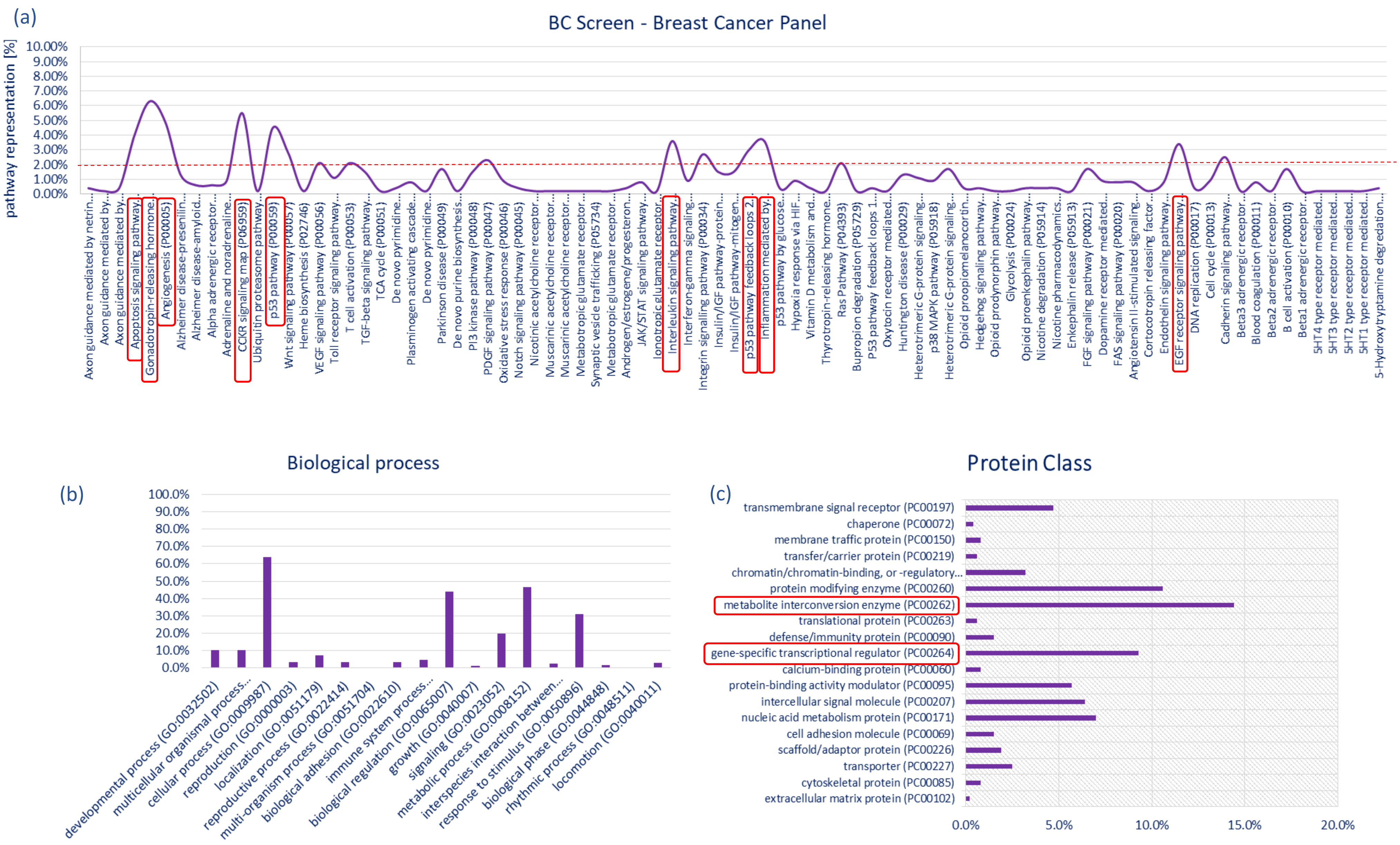

(a)
BC Screen - Breast Cancer Panel
pathway representation [%]
10.00%
9.00%
8.00%
7.00%
6.00%
5.00%
4.00%
3.00%
2.00%
1.00%
0.00%
Axon guidance mediated by netrin...
Axon guidance mediated by...
Axon guidance mediated by...
Apoptosis signaling pathway...
Gonadotropin-releasing hormone...
Angiogenesis (P00005)
Alzheimer disease-presenilin...
Alzheimer disease-amyloid...
Alpha adrenergic receptor...
Adrenaline and noradrenaline...
CCKR signaling map (P06959)
Ubiquitin proteasome pathway...
p53 pathway (P00059)
Wnt signaling pathway (P00057)
Heme biosynthesis (P02746)
VEGF signaling pathway (P00056)
Toll receptor signaling pathway...
T cell activation (P00053)
TGF-beta signaling pathway...
TCA cycle (P00051)
De novo pyrimidine...
Plasminogen activating cascade...
De novo pyrimidine...
Parkinson disease (P00049)
De novo purine biosynthesis...
PI3 kinase pathway (P00048)
PDGF signaling pathway (P00047)
Oxidative stress response (P00046)
Notch signaling pathway (P00045)
Nicotinic acetylcholine receptor...
Muscarinic acetylcholine receptor...
Muscarinic acetylcholine receptor...
Metabotropic glutamate receptor...
Synaptic vesicle trafficking (P05734)
Metabotropic glutamate receptor...
Androgen/estrogene/progesteron...
JAK/STAT signaling pathway...
Ionotropic glutamate receptor...
Interleukin signaling pathway...
Interferon-gamma signaling...
Integrin signalling pathway (P00034)
Insulin/IGF pathway-protein...
Insulin/IGF pathway-mitogen...
p53 pathway feedback loops 2...
Inflammation mediated by...
p53 pathway by glucose...
Hypoxia response via HIF...
Vitamin D metabolism and...
Thyrotropin-releasing hormone...
Ras Pathway (P04393)
Bupropion degradation (P05729)
P53 pathway feedback loops 1...
Oxytocin receptor mediated...
Huntington disease (P00029)
Heterotrimeric G-protein signaling...
p38 MAPK pathway (P05918)
Heterotrimeric G-protein signaling...
Opioid proopiomelanocortin...
Hedgehog signaling pathway...
Opioid prodynorphin pathway...
Glycolysis (P00024)
Opioid proenkephalin pathway...
Nicotine degradation (P05914)
Nicotine pharmacodynamics...
Enkephalin release (P05913)
FGF signaling pathway (P00021)
Dopamine receptor mediated...
FAS signaling pathway (P00020)
Angiotensin II-stimulated signaling...
Cortocotropin releasing factor...
Endothelin signaling pathway...
EGF receptor signaling pathway...
DNA replication (P00017)
Cell cycle (P00013)
Cadherin signaling pathway...
Beta3 adrenergic receptor...
Blood coagulation (P00011)
Beta2 adrenergic receptor...
B cell activation (P00010)
Beta1 adrenergic receptor...
5HT4 type receptor mediated...
5HT3 type receptor mediated...
5HT2 type receptor mediated...
5HT1 type receptor mediated...
5-Hydroxytryptamine degredation...
(b)
Biological process
100.0%
90.0%
80.0%
70.0%
60.0%
50.0%
40.0%
30.0%
20.0%
10.0%
0.0%
developmental process (GO:0032502)
multicellular organismal process...
cellular process (GO:0009987)
reproduction (GO:0000003)
localization (GO:0051179)
reproductive process (GO:0022414)
multi-organism process (GO:0051704)
biological adhesion (GO:0022610)
immune system process...
biological regulation (GO:0065007)
growth (GO:0040007)
signaling (GO:0023052)
metabolic process (GO:0008152)
interspecies interaction between...
response to stimulus (GO:0050896)
biological phase (GO:0044848)
rhythmic process (GO:0048511)
locomotion (GO:0040011)
(c)
Protein Class
transmembrane signal receptor (PC00197)
chaperone (PC00072)
membrane traffic protein (PC00150)
transfer/carrier protein (PC00219)
chromatin/chromatin-binding, or -regulatory...
protein modifying enzyme (PC00260)
metabolite interconversion enzyme (PC00262)
translational protein (PC00263)
defense/immunity protein (PC00090)
gene-specific transcriptional regulator (PC00264)
calcium-binding protein (PC00060)
protein-binding activity modulator (PC00095)
intercellular signal molecule (PC00207)
nucleic acid metabolism protein (PC00171)
cell adhesion molecule (PC00069)
scaffold/adaptor protein (PC00226)
transporter (PC00227)
cytoskeletal protein (PC00085)
extracellular matrix protein (PC00102)
0.0%
5.0%
10.0%
15.0%
20.0%

Figures S1-S7. *Classification of proteins and pathways.* Quantigene (3 panels: cancer pathway, stress & toxicity and epigenetic and chromatin modification) (S1-S3), The Attagene-cis-factorial (S4), nCounter Pan Cancer (S5), Trusight Pan Cancer (S6), BC Screen model (S7) transcriptomics-based assays were described using the PANTHER (Protein ANalysis THrough Evolutionary Relationships) Classification System [http://www.pantherdb.org/; (Mi et al. NAR 2013; Thomas et al., Genome Research 2003)]. PANTHER Analysis allowed a preliminary classification of proteins (and their genes) of each single transcriptomics-based assay. a) Percent representation of pathways; b) Percent representation of biological processes, based on GO annotations. c) Percent representation of protein classes. Red boxes identify most represented pathways, biological processes or protein classes within each single assay.
